# Bioactivity and nutritional quality of nutgall (*Rhus semialata* Murray), an underutilized fruit of Manipur

**DOI:** 10.3389/fnut.2023.1133576

**Published:** 2023-06-05

**Authors:** Thangjam Surchandra Singh, Pintubala Kshetri, Asem Kajal Devi, Pangambam Langamba, Keishing Tamreihao, Heikham Naresh Singh, Romila Akoijam, Tania Chongtham, Chingakham Premabati Devi, Tensubam Basanta Singh, Sonia Chongtham, Yumnam Prabhabati Devi, Aparna Kuna, Soibam Giri Singh, Susheel Kumar Sharma, Anup Das, Subhra Saikat Roy

**Affiliations:** ^1^ICAR Research Complex for NEH Region, Manipur Centre, Imphal, India; ^2^YK College, Wangjing, Manipur, India; ^3^St. Joseph College, Ukhrul, Manipur, India; ^4^Quality Control Laboratory, Professor Jayashankar Telangana State Agricultural University, Hyderabad, India; ^5^ICAR-Indian Agricultural Research Institute, New Delhi, India; ^6^ICAR Research Complex for Eastern Region, Tripura Centre, Lembucherra, India; ^7^ICAR-Central Citrus Research Institute, Nagpur, Maharashtra, India

**Keywords:** *Rhus semialata* Murray, nutrients, amino acids, fatty acids, antioxidant, antidiabetic, xanthine oxidase inhibition

## Abstract

**Introduction:**

Underutilized fruits plays a significant role in socio economic, cultural, nutritional and ethnomedicinal status of tribal people. However, scientific studies on the nutritional and other pharmaceuticals/biological activities of these fruits are meagre. Hence, the present study dealt with the quantification of nutritional quality and deciphering the bioactivity of nutgall (*Rhus semialata* Murray syn. *Rhus chinensis* Mill.), an underutilized fruit crop mainly found in foothill tracks of Eastern Himalaya, India, China, Japan, Korea and other South East Asian countries.

**Methods:**

The *Rhus semialata* Murray fruits were collected from five different locations in Purul sub-division, Senapati district, Manipur, India. The nutritional composition of the fruit pulp was analysed. Further the fruit pulp was extracted in methanol and water. The methanol and water extracts were studied for bioactivity properties such as antioxidant, antihyperglycemic, antihypertensive, antihyperuricemia, anti-tyrosinase, and antimicrobial activity.

**Results and discussion:**

The fruit was rich in essential fatty acids. The presence of linoleic and oleic acids, along with traces of docosahexaenoic acid and eicosapantaenoic acid, revealed the potential food value of the fruit. 59.18% of the total amino acid composition of the protein present was constituted by essential amino acids. The IC_50_ value of methanolic extract (MExt) and Water extract (WExt) of the fruit were recorded as 4.05 ± 0.22 and 4.45 ± 0.16 μg/mL, respectively, in the DPPH assay and 5.43 ± 0.37 and 11.36 ± 2.9 μg/mL, respectively, in the ABTS assay as compared to Ascorbic acid (3 and 5.4 μg/mL in DPPH and ABTS assay, respectively). The CUPRAC assay also showed a high antioxidant potential of MExt and WExt (1143.84 ± 88.34 and 456.53 ± 30.02 mg Ascorbic Acid Equivalent/g, respectively). MExt and WExt of the fruit were more active against α-glucosidase (IC_50_ of 1.61 ± 0.34 and 7.74 ± 0.54 μg/ mL, respectively) than α-amylase enzyme (IC_50_ 14.15 ± 0.57 and 123.33 ± 14.7 μg/mL, respectively). In addition, the methanolic fruit extract showed low to moderate pharmacological potential in terms of antihypertensive (Angiotensin converting enzyme-I inhibition), antihyperuricemia (xanthine oxidase inhibition), anti-tyrosinase, and antimicrobial activity. The IC_50_ values of angiotensin-converting enzyme I inhibition, xanthine oxidase inhibition and tyrosinase inhibition were recorded as 13.35 ± 1.21 mg/mL, 93.16 ± 4.65 mg/mL, and 862.7 ± 12.62 μg/mL, respectively. The study evidently indicates that nutgall fruit is a potential source of phytonutrients, bestowed with commercially exploitable, multifaceted health benefits

## Introduction

Fruits are known to be rich source of antioxidants, soluble fiber, nutrients, phenols, flavones, vitamins, and minerals. Low intake of fruits is connected with unhealthy diets and several chronic illnesses such as aging, cancer, and cardiovascular disease (1). Edible wild fruits or underutilized fruits serve an important role in augmenting people’s diets, particularly in rural and tribal areas. Underutilized fruit crops (a neglected and hidden resource) are referred to the fruit crops that have potential but are not farmed commercially on a large scale (2). Because of their affordability and easy accessibility in ethnic food baskets, they have the potential to play a prospective role in attaining nutritional security and eradicating malnutrition, particularly in disadvantageous areas. Underutilized fruits are also wonderful source of phytonutrients and inadvertently contribute to control “hidden hunger” among the underprivileged communities.

Recognizing the significance of underutilized food crops, the International Centre for Underutilized Crops (ICUC) promotes their cultivation, usage, and marketing. These crops are also important for sustaining floral biodiversity (3). Furthermore, less economic input is required in cultivation because these crops require minimal maintenance and do not require much in the way of agro-inputs such as irrigation, fertilizers, pesticides, and so on. They are also well adapted to local agro-ecology and possess systemic resistance against biotic and biotic stresses.

Underutilized fruits are not only high in nutrients, but they are also believed to have a wide array of therapeutic properties such as antiaging, antibacterial, antidiabetic, anti-inflammatory, antihypertensive, cardioprotective, hypolipidemic, and anti-tyrosinase properties (4). Singh et al. (5), for example, revealed that *Viburnum mullaha* (Buch.-Ham. Ex D. Don), an underutilized wild edible fruit of the Indian Himalayan region, had anti-tyrosinase and antiaging activity. Antidiabetic properties have been identified in underutilized fruits found in Indian subcontinent such as *Syzygium cumini* L. (6) and *Spondias mangifera* (7). Similarly, Ibrahim et al. (8) found that five underutilized Nigerian fruits, *Ziziphus spinachristi, Ziziphus mairei, Parkia biglobosa, Detarium microcarpum*, and *Parinari macrophylla* inhibited intestinal maltase and sucrase in rats.

Consumption of fruit has been shown to significantly reduce blood pressure. Some underutilized indigenous fruits (*Trapa bispinosa, Phoenix sylvestris, Cicca acida, Achras sapota*, and *Averrhoa carambola*) have been shown to inhibit angiotensin converting enzyme I (9). Antibacterial activity has been observed for *Syzygium calophyllifolium* (10), *Garcinia gummi-gutta* (L.) N. Robson, and *Artocarpus lacucha* Buch.-Ham (11). Since time immemorial, traditional healers, as well as ayurveda, unani, and homeopathy, have employed many of the underutilized indigenous fruits or plant parts as medication (12). Because of these reasons, underutilized fruits have the potential to be employed as nutraceuticals and functional foods.

The Himalayan range is the home to the world’s largest agro-ecosystem and the rarest crop biodiversity (13). In India, the Himalayan region is a rich reservoir of many underutilized fruit crops. However, comprehensive scientific research on nutritional and other pharmaceutical potential of these fruit crops has been sporadically undertaken. Nutgall (*Rhus semialata* Murray syn. *R. chinensis* Mill., *R. javanica* Linn.) is one such underutilized fruit crop found in the hills and valley regions of North East Indian Himalayan region. It is also wildly distributed in China, Japan, Java, North Korea, Malaysia, Indo-China Peninsula, Indonesia, Europe, and USA (14–16). The genus *Rhus* belongs to the Anacardiaceae family and consists of more than 250 species distributed in the tropics, subtropics and temperate regions. The fruits are edible and have a sharp, acidic taste. The fruits are spheroidal and drupe, slightly flattened, and are about 4–5 mm in diameter. It is separated into pulp and seed.

The fruits are nutrient-dense, including macronutrients, micronutrients, vitamins (vitamin C, B5, B6, and B9), and phytochemicals such as polyphenols, carotenoids, flavonoids, and others. It also includes vital fatty acids and amino acids, confirming the its nutritional excellence (17). Aside from its nutritional value, the fruit also demonstrated medicinal qualities. The infusion of Rhus fruits, for example, is traditionally used in traditional medicine practices to treat fungal, bacterial, and protozoal diseases (diarrhea and dysentery), food poisoning, and allergies in both people and animals (18). According to Bose et al. (19), the methanolic fruit extract of *R. semialata* demonstrated anti-diarrheal efficacy in rats by reducing gastrointestinal propulsion and fluid secretion. The fruit has also been made into digestion pills (20).

Given this backdrop, the current study sought to investigate the nutritional and biological activity of *Rhus semialata* Murray, an underutilized fruit collected from Manipur, India. There are reports in the literature on the nutritional (carbohydrate, fat, protein, mineral, etc.) and some medicinal qualities of this fruit. However, the current study focused on other aspects of the nutritional profile, such as amino acid content, important fatty acids, and so on. Furthermore, biological properties such as antioxidant, antihypertension, anti-hyperglycemic, antityrosinase, anti-gout, antibacterial activities and cytotoxicity were also assessed.

## Methodology

### Collection of samples and processing

Matured fruits were collected from five different locations in Purul sub-division, Senapati district, Manipur, India (25.37 N, 94.22E, 1,620 m above msl). About 2 kg of fresh fruit was collected from five different plants (400 g each plant) in each location and removed any unwanted non-fruit portions then dried overnight in the oven at 40–45°C. The dried fruit was hand crushed (using a sterile hand glove) to detach the pulp from the seed. Then the pulp was separated by winnowing and ground to a fine powder and stored in an air tide container at 4°C until further used.

### Preparation of methanol extract (MExt)

Ten gram of fruit pulp powder was soaked in methanol (100 mL) at room temperature for 24 h with shaking at 180 rpm (Spinix orbital shaker, Tarson). Extraction was repeated for 48 and 72 h and filtered through Whatman no. 1 filter paper followed by drying at 45°C under low pressure (Rotatory evaporator, IKA, Germany). The dried sample was stored at −20°C until used for biological activity assay.

### Preparation of water extract (WExt)

Ten gram of fruit pulp powder was soaked in deionized water (50 mL) at room temperature for 3 h with shaking at 180 rpm (Spinix orbital shaker, Tarson) then centrifuged at 10,000 rpm for 10 min. Extraction was repeated with the residue for another hour. The supernatants were pooled in a 250 mL flask and kept at −20°C for overnight. The sample was lyophilized at −80°C. The lyophilized sample was stored at −20°C until further use for biological activity assay.

### Proximate and nutritional content

For proximate and nutrient analysis, fruit pulp sample was used. The parameters for proximate analysis *viz.* moisture (Method No. 934.01), crude protein (Method No. 2001.11), fat (Method No. 2003.05), total carbohydrate, ash content (Method No. 942.05) and micronutrients (Method No. 975.03) were determined according to AOAC (21). Total carbohydrate content was calculated by difference (22). The vitamin C was determined by the spectrophotometric method (23). For determination of ash and micronutrient analysis, 1 g dried sample was taken in a pre-weighed 15 mL crucible and digested at 550°C for 4 h in a muffle furnace till white residue remained. The residue was weighed and used for ash determination. For micronutrient analysis, the residue was dissolved in 10 mL HCl (2 N) using a glass rod and then filtered in Whatman No. 42 (ashless). The volume was made upto 50 mL using distilled water. The micronutrients (Ca, Fe, Zn, Mn, and Cu) were measured in an atomic absorption spectrophotometer (AAS) (Perkin Elmer, United States). Total phosphorous (P) was measured by spectrophotometer (450 nm) using ammonium vanadate-ammonium molybdate reagent and potassium was measured in a flame photometer (BioEra, India). The values were expressed in mg/100 g sample.

### Amino acid analysis

The amino acid profile of *Rhus semialata* Murray fruit sample was determined using HPLC (Agilent, United States) equipped with a diode array and multiple wavelength detector. The fruit sample was hydrolyzed with 6 N HCl, and the hydrolyzed peptide samples were derivatized with OPA (o–phthalaldehyde for primary amino acids) and FMOC (9–fluorenylmethyl chloroformate for secondary amino acids) as reported by Sahoo et al. (24). The derivatized samples were analyzed for amino acid composition using a Zorbax Eclipse–AAA column (250 mm × 4.6 mm, L × ID, particle size 5 μm) (Agilent Technologies, Santa Clara, CA). The amino acid composition was expressed as the percent amino acid of the total protein content of the analyzed sample.

### Fatty acid analysis

The total fat content of the moisture-free sample was extracted in Gerhardt Soxtherm fat analyzer [(21), Method No. 2003.06; (25)]. Fatty acids were analyzed by AOAC (21) (Method No. 996.06) and Shaik et al. (25) methods. The isolated fat was trans-esterified using 0.5 M methanolic KOH to form fatty acid methyl esters (FAME), which were then estimated by Gas Chromatograph (7890B of Agilent Technologies) equipped with a flame ionization detector and an Agilent-DB-FFAP column (nitroterephthalic-acid-modified polyethylene glycol (PEG) of high polarity for the analysis of volatile fatty acids). The temperature of the column was maintained at an initial temperature of 100°C for 5 min, then raised up to 240°C at the rate of 4°C/min. Nitrogen was used as carrier gas at a column flow rate of 1.0 mL/min. The detector temperature was maintained at 280°C. Standards used were 47,885*-U* Supelco^®^ 37 Component FAME Mix (10 mg/mL in methylene chloride). Sample fatty acid composition was compared with standard fatty acid composition, and percentages were calculated by normalization of peak areas.

### Antioxidant assay

#### 2,2-diphenyl 1- picrylhydrazyl (DPPH) assay

0.1 mL of each of the five different concentrations of the extract was mixed with 1.9 mL DPPH reagent prepared in methanol (A_517_ = 1.1 + 0.01). The mixture was incubated for 30 min in the dark and then read at 517 nm. The radical scavenging activity (RSA) of each extract was calculated as follows (26).


$$\text{RSA}\%=\left(A_{\text{control}}-A_{\text{sample}}\right)/A_{\text{control}}\;X100\%$$

From the RSA%, 50 inhibitory concentration (IC_50_) was calculated and expressed in μg/mL. IC_50_ is defined as the minimum amount of the extract required to scavenge half of the initial stable free radical in 30 min in 1 mL of reaction in the dark.

#### 2,2′-azino-bis (3-ethylbenzothiazoline-6-sulphonic acid) (ABTS) assay

ABTS radical scavenging activity was performed as described by Singh et al. (26). The reaction mixture containing 0.1 mL of extract was mixed with 1.9 mL ABTS radical (A_734_ = 1.0 ± 0.01) then incubated for 30 min in the dark, and the absorbance was read at 734 nm. RSA and IC_50_ of the extract were calculated as described above.

#### Ferric reducing antioxidant power (FRAP) assay

0.1 mL of each extract dissolved in methanol was reacted with 1.9 mL FRAP reagent and incubated for 5 min and the absorbance was read at 593 nm. FeSO_4_ solution (5–30 μg/mL) was used as standard and ferric reducing antioxidant power was expressed as mM ferrous equivalent per gram of the extract (mM Fe Eq/g Ext.) (26).

#### CUPRAC (cupric ion reducing antioxidant capacity) assay

0.5 mL sample was mixed with 0.5 mL copper II chloride (10 mM) in a tube. 0.5 mL neocuporine solution was added, and followed by 0.5 mL ammonium acetate solution (1 M). The reaction mixture was incubated for 30 min at room temperature and the color developed was read at 450 nm. Ascorbic acid was used as a standard. Cupric ion reducing antioxidant capacity was expressed in milligram ascorbic acid equivalent per gram of extract (mg AAE/g extract) (27).

#### Phenolic and flavonoid assay

The total phenolic and flavonoid content of the fruit extract was analyzed by Folin Ciocalteu and aluminum chloride methods, respectively described in Singh et al. (26). Phenolic content was expressed in milligram of gallic acid equivalent per gram of extract (mg GAE/g), whereas flavonoid content was expressed in milligram of quercetin equivalent per gram of extract (mg QE/g).

### Pharmaceutical property

#### Antihyperglycemic activity

##### α-amylase inhibitory assay

0.1 mL of the sample extract (5–50 μg) was taken in a 2 mL tube and mixed with 0.1 mL enzyme (1 U/mL, porcine pancreas), incubated at 37°C for 20 min. The enzyme reaction was initiated by the addition of 0.2 mL of starch solution (1%) and continued for 3 min at 37°C. After 3 min, 0.2 mL dinitrosalicylic acid (DNSA) reagent was added and kept incubated at 80°C, followed by 1.4 mL of distilled water. For the control reaction, instead of sample, the buffer was added, and a blank was prepared by the addition of DNSA before the addition of the enzyme. The color complex was read at 540 nm. Acarbose (1–10 μg) was used as a standard positive control. From the percentage inhibition, IC_50_ was calculated (28).

##### α-glucosidase inhibitory assay

0.1 mL of the sample extract (0.1–50 μg) was taken in a 2 mL tube and mixed with 0.2 mL enzyme (0.5 U/mL, α-glucosidase from *S. cerevisiae*), incubated at 37°C for 10 min. To the mixture solution, 0.1 mL 4-nitrophenyl α-D-glucopyranoside (2.5 mM) was added and incubated for 5 min at 37°C. The reaction was terminated by the addition of 0.2 mL sodium carbonate (20%) followed by the addition of 1 mL water. The color complex was measured at 405 nm against a reagent blank (29).

##### Xanthine oxidase inhibitory assay

0.1 mL sample of different concentrations (50–300 μg) was taken in a tube and the volume was made up to 0.6 mL with buffer (0.05 M, pH 7.5), and 0.1 mL enzyme (0.2 U/mL), followed by incubation for 15 min at 37°C. In the reaction mixture, 0.2 mL substrate (xanthine; 0.15 mM) was added and then incubated for 30 min at 37°C. After 30 min, 0.2 mL HCl (0.5 M) was added and then read at 293 nm against a reagent blank. Allopurinol was used as a positive control (30).

##### ACE (angiotensin-converting enzyme I) inhibitory assay

0.01 mL of enzyme was added to a tube containing 0.140 mL sample prepared in buffer (pH 8.3) and 0.05 mL sodium chloride (300 μM). To the reaction mixture, 0.5 mL substrate (hipuryl-his-leu) was added and incubated for 30 min. After 30 min, the reaction mixture was kept in a boiling water bath for 10 min. After cooling, absorbance was read at 385 nm. Captopril was used as a positive control (30).

##### Antityrosinase assay

0.340 mL of different concentrations of the sample (0.5–2 mg) was mixed with 0.02 mL enzyme (mushroom tyrosinase) and 0.360 mL substrate (16 mM DOPA) in a tube and then read at 480 nm spectrophotometrically for 3 min for every 30 s. For control (100% activity), instead of sample, 0.340 mL buffer was used. Kojic acid was used as a positive control (30).

##### Antibacterial assay

The Kirby-Bauer disk diffusion method (31) was used for the evaluation of the antibacterial activity of MExt. Four test organisms, *viz.*; *Salmonella typhimurium* (ATTC13311), *Escherichia coli* (MTCC 739), *Bacillus subtilis* (MTTCC 121), *Staphylococcus aureus* (ATCC 1026), were grown in Mueller-Hinton broth (MHB) at 37°C and 160 rpm and brought to its exponential phase. The sample was prepared by dissolving the MExt in 50% DMSO (at 25 mg/mL w/v concentration) and filtering through a (0.22 μm) syringe filter. Hundred microliter of each bacterial strain, equivalent to 0.5 McFarland turbidity were spread over sterile Mueller-Hinton agar (MHA) plate using a sterile swab. A 20 μL sample (0.5 mg) impregnated in a 6 mm sterile disc was seeded on the freshly spread MHA plates and incubated for 24 h at 37°C. Chloramphenicol 0.025 mg was used as a positive control. One reagent control containing the buffer and solvent used was also included in the experiment. The zone of inhibition was recorded using a Vernier caliper.

##### *In vitro* cytotoxicity assay

The cytotoxic activity of MExt was investigated using Sulforhodamine B colorimetric (SRB) assay against HeLa (human cervical cancer cell line). The cell was maintained in a Dulbecco’s Modified Eagle Medium (DMEM). The cells were subjected to different concentrations of the extracts (5–100 μg/mL) for 48 h. Doxorubicin (5–100 μg/mL) was used as a positive control. The monolayer cells were then fixed with 10% Trichloroacetic acid (TCA) for 1 h at 40°C followed by washing with water. After drying, the cell plates were stained with SRB for 30 min and then washed with 1% acetic acid. The SRB dye bound to the protein was then dissolved by adding 10 mM Tris base (pH 10.5). The SRB dye released was quantified by taking absorbance at 510 nm in Varioskan Flash Multimode Reader (Thermo Fisher Scientific, United States) (32).

##### Statistical analysis

All the assays were performed with three replications, and each replication had 3 triplicates. The data are presented as mean ± standard deviation (SD). The IC_50_ values of assays were calculated using the Prism software (GraphPad, United States).

## Results

### Nutritional analysis

The nutritional contents of the *Rhus semialata* Murray are presented in Table 1. The fruit pulp was mainly comprised of moisture (11.32 ± 0.45%), total carbohydrate (59.2 ± 1.68%), crude fat (19.21 ± 0.76%), ash (4.02 ± 0.13%), and crude protein (4.25 ± 0.04%). The fruit also contained vitamins and minerals. The vitamin C content of the fruit was 95.24 ± 0.15 mg/100 g while the nutrients such as P, Fe, Mg, Zn, Cu, and Mn were 218 ± 6.8, 19.92 ± 0.2, 14.96 ± 1.2, 2.43 ± 0.4, 1.19 ± 0.08, and 0.93 ± 0.06 mg/100 g, respectively.

**Table 1:** Proximate constituent of *Rhus semialata* fruit pulp.

Parameter	g/100 g
Moisture	11.32 ± 0.45
Crude protein	4.25 ± 0.04
Crude fat or oil	19.21 ± 0.76
Total carbohydrate	59.20 ± 1.68
Ash	4.02 ± 0.13
Vitamin C	95.24 ± 0.15
**Micronutrient**	**mg/100 g**
P	218.00 ± 6.8
Fe	19.92 ± 0.2
Mg	14.96 ± 1.2
Zn	2.43 ± 0.4
Cu	1.19 ± 0.08
Mn	0.93 ± 0.06

### Amino acid composition

A total of 17 amino acids were detected in the fruit sample (Table 2). Of these, the essential amino acids, (EAAs) *viz.* histidine, isoleucine, leucine, lysine, methionine, phenylalanine, threonine and valine, constituted 59.18% of the total amino acid composition. Among the EAA, threonine (27.21%) was the highest, followed by lysine (14.64%) and leucine (4.71%), while isoleucine (1.92%) was the lowest. The two EAAs, threonine and lysine, constituted 41.85% of the total amino acid content. The non-essential amino acid content was in the range of 0.71–8.64%. Among the nonessential amino acids, glutamate (8.64%) was recorded as the highest followed by cysteine (7.80%), serine (5.63%), and Arginine (5.43%), while glycine (1.12%) and tyrosine (0.71%) were the lowest.

**Table 2:** Amino acid composition of *Rhus semialata* Murray fruit pulp.

Name of the amino acid	% composition
Threonine	27.21
Lysine	14.64
Glutamine	8.64
Cystine	7.80
Serine	5.63
Arginine	5.43
Alanine	4.71
Leucine	4.71
Hydroxy proline	3.64
Aspartate	3.11
Valine	2.88
Phenylalanine	2.82
Methionine	2.75
Histidine	2.25
Isoleucine	1.92
Glycine	1.12
Tyrosine	0.71

### Fatty acid composition

The total fat content of the studied fruits was constituted by three different groups of fatty acids: polyunsaturated fatty acid (PUFA) (50.62%), saturated fatty acid (SFA) (35.13%), and monounsaturated fatty acid (MUFA) (12.54%), while 1.72% of fatty acids were unknown fatty acids (Table 3). The PUFA included 8 fatty acids, *viz.*, Linoleic acid methyl ester (C18:2n6c), y-linolenic acid methyl ester (C18:3n6), α-linolenic acid methyl ester (C18:3n), Cis-11,14,17 eicosatrienoic acid methyl ester (C20:3n3), Arachidonic acid methyl ester (C20:4n6), Cis-5,8,11,14,17- eicopentaenoic acid methyl ester (C20:5n3), Cis-13,16-docosadienoic methyl ester (C22:2) and Cis-4,7,10,13,16,19-docosahexaenoic acid methyl ester (C22:6n3). Saturated fatty acids (SFA) were obtained in the ranges of 0.08–28.46% of total fatty acids. The most abundant saturated fatty acid was palmitic acid (28.46%), followed by steric acid (C18:0) (2.92%) and butyric acid (2.38). Monounsaturated fatty acids were obtained in the range of 0.33–11.53%. The highest MUFA was recorded as oleic acid (11.15%), and followed by nervoinic acid (0.35%).

**Table 3:** Fatty acid composition of *Rhus semialata* Murray fruit pulp.

Names of fatty acid	% composition
**Saturated fatty acid (SFA)**	**35.13**
Butyric acid (C4:0)	2.38
Myristic acid (C14:0)	0.15
Palmitic acid (C16:0)	28.46
Heptadecanoic acid (C17:0)	0.09
Stearic (C18:0)	2.92
Arachidic acid (C20:0)	0.20
Heneicosanoic acid (C21:0)	0.13
Behenic acid (C22:0)	0.72
Lignoceric acid (C24:0)	0.08
**Monounsaturated fatty acid (MUFA)**	**12.54**
Palmitoleic acid (C16:1)	0.33
Oleic acid (C18:1n9c)	11.53
Cis-11 eicosenoic acid (C20:1n9c)	0.33
Nervoinic acid (C24:1n9c)	0.35
**Poly unsaturated fatty acids (PUFA)**	**50.62**
Linoleic acid (C18:2n6c)	46.68
y-linolenic acid (C18:3n6)	2.61
α-linolenic acid (C18:3n)	0.49
Cis-11,14,17, eicosatrienoic acid (C20:3n3)	0.15
Arachidonic acid methyl ester (C20:4n6)	0.13
Cis-5,8,11,14,17- eicopentaenoic acid (C20:5n3)	0.27
Cis-13,16-docosadienoic acid (C22:2)	0.05
Cis-4,7,10,13,16,19-docosahexaenoic acid (C22:6n3)	0.24
Unknown	**1.72**

The bold value indicates the total value of the of the major fatty acid group SFA, MUFA, PUFA etc.) for example the total saturated fatty acid content is 35.13 which is further composed of individual fatty acids buytyric acid…… lignocric acid all the values of the individual fatty acid content will give the total value (i.e 35.13).

### Antioxidant activity

The fruit exhibited significant free radical scavenging activity in the ABTS and DPPH assay. In the ABTS assay, the IC_50_ value for MExt and WExt was exhibited as 5.43 ± 0.37 μg/mL and 11.36 ± 2.91 μg/mL, respectively. While the IC_50_ value of standard ascorbic acid was recorded as 3.0 μg/mL. In the DPPH assay, the IC_50_ value for MExt and WExt was found to be 4.05 ± 0.12 μg/mL and 4.45 ± 0.06 μg/mL, respectively. While the IC_50_ value of standard ascorbic acid was recorded as 5.40 μg/mL.

The fruit extract also exhibited ferric and cupric ion-reducing antioxidant activity. The FRAP assay revealed that the MExt and WExt could reduce 4.89 ± 0.68 mM of Fe^+3^/g Ext. and 1.23 ± 0.07 mM Fe^+3^/g Ext, respectively. In the CUPRAC assay, the antioxidant activity of the fruit exhibited 1134.84 ± 88.06 mg AAE/g and 456.53 ± 30.02 mg AAE/g for MExt and WExt, respectively (Table 4).

**Table 4:** Total phenolic, flavonoid content and antioxidant activity.

Antioxidant assay	Methanol extract	Water extract	Vitamin C (positive control)
ABTS (IC_50_ μg/mL)	5.43 ± 0.37	11.36 ± 2.91	3.00 ± 0.40
DPPH (IC_50_ μg/mL)	4.05 ± 0.12	4.45 ± 0.06	5.40 ± 0.30
FRAP (mM Fe Eq/g Ext)	4.89 ± 0.68	1.23 ± 0.07	–
CUPRAC (mg AAE/g Ext)	1134.84 ± 88.06	456.53 ± 30.02	–
Total phenolic (mg GAE/g)	310.37 ± 3.10	255.46 ± 4.40	–
Total flavonoid (mgQE/g Ext)	110.27 ± 20.23	83.02 ± 4.20	–

### Phenolic and flavonoid content

The phenolic content of MExt and WExt fruit extract was found to be 310.37 ± 3.1 and 255.46 ± 4.4 mg GAE/g extract, respectively. Whereas the flavonoid content of MExt and WExt fruit extract were recorded as 110.27 ± 20.23 and 83.02 ± 4.2 mg QE/g extract, respectively (Table 4).

### Pharmaceutical activity

#### Antihyperglycemic activity

The fruit extracts demonstrated anti-hyperglycaemic activity by inhibiting α-amylase and α-glucosidase enzyme (Table 5). The IC_50_ value of MExt and WExt was found to be 14.15 ± 1.57 μg/mL and 123.33 ± 14.7 μg/mL, respectively. While the IC_50_ of standard acarbose was recorded as 6.12 ± 0.68 μg/mL. The fruit extract also inhibited the α-glucosidase enzyme. The IC_50_ value of MExt and WExt was found to be 1.61 ± 0.34 and 7.74 ± 0.54 μg/mL while the standard acarbose was 404 ± 17.24 μg/mL (Table 5).

**Table 5:** Bioactivity of *Rhus semialata* Murray fruit pulp.

Bioactivity	IC_50_ μg/mL		
	Methanol extract	Water extract	Positive control
α-amylase inhibitory	14.15 ± 1.57	123.33 ± 14.70	Acarbose: 6.12 ± 0.68
α-glucosidase inhibitory	1.61 ± 0.34	7.74 ± 0.54	Acarbose: 404.00 ± 17.24
Xanthine oxidase inhibitory	93.16 ± 4.65	192.20 ± 1.90	Allopurinol 77.00 ± 0.08
ACE inhibitory	13.35 ± 1.21	34.57 ± 3.56	Captopril: 0.005 ± 0.0003
Tyrosinase inhibitory	862.70 ± 12.62	1026.00 ± 38.00	Kojic acid: 28.50 ± 2.00

#### Xanthine oxidase inhibitory assay

The methanolic fruit extract was found to inhibit the xanthine oxidase enzyme. The IC_50_ of MExt was 93.16 ± 4.65 μg/mL and 192.2 ± 1.9 μg/mL, respectively, while the standard drug allopurinol was 77 ± 0.085 μg/mL (Table 5).

#### Angiotensin-converting enzyme-I (ACE) inhibitory assay

The fruit extract showed inhibitory activity in Angiotensin-converting enzyme-I. The IC_50_ value of MExt and WExt was 13.35 ± 1.21 μg/mL and 34.57 ± 3.56 μg/mL, whereas the standard captopril was 0.00458 ± 0.0 μg/mL (Table 5).

#### Antityrosinase activity

The fruit extract displayed inhibitory activity against the tyrosinase enzyme. The IC_50_ value of MExt and WExt was 862.7 ± 12.62 μg/mL and 1,026 ± 38 μg/mL, respectively, whereas the IC_50_ of the standard kojic acid was found to be 27.04 ± 1.6 μg/mL (Table 5).

#### Antibacterial assay

**Figure 1:**
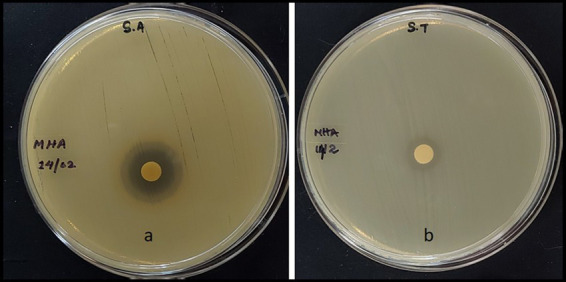
Antibacterial activity of methanolic fruit extract of *R. semialata* Murray **(a)** *Staphylococcus aureus* **(b)** *Salmonella typhi.*

The MExt also exhibited promising antimicrobial activity against *Salmonella typhi* and *Staphylococcus aureus*. However, it did not inhibit the growth of *Escherichia coli* and *Bacillus subtilis* organisms. The inhibition zone of *Salmonella typhi* and *Staphylococcus aureus were* 9.5 ± 0.4 mm and 12.39 ± 0.07 mm, respectively (Figure 1 and Table 6).

**Table 6:** Antimicrobial activity of *Rhus semialata* Murray fruit pulp.

Sample	Inhibition zone (mm)			
	*Bacillus subtilis*	*Eschericia coli*	*Salmonella typhi*	*Staphylococcus aureus*
Methanolic extract	–	–	9.50 ± 0.40	12.39 ± 0.07
Chloramphenicol	30.20 ± 1.60	28.00 ± 2.20	30.00 ± 2.00	29.00 ± 1.00

#### *In vitro* cytotoxicity assay

**Figure 2:**
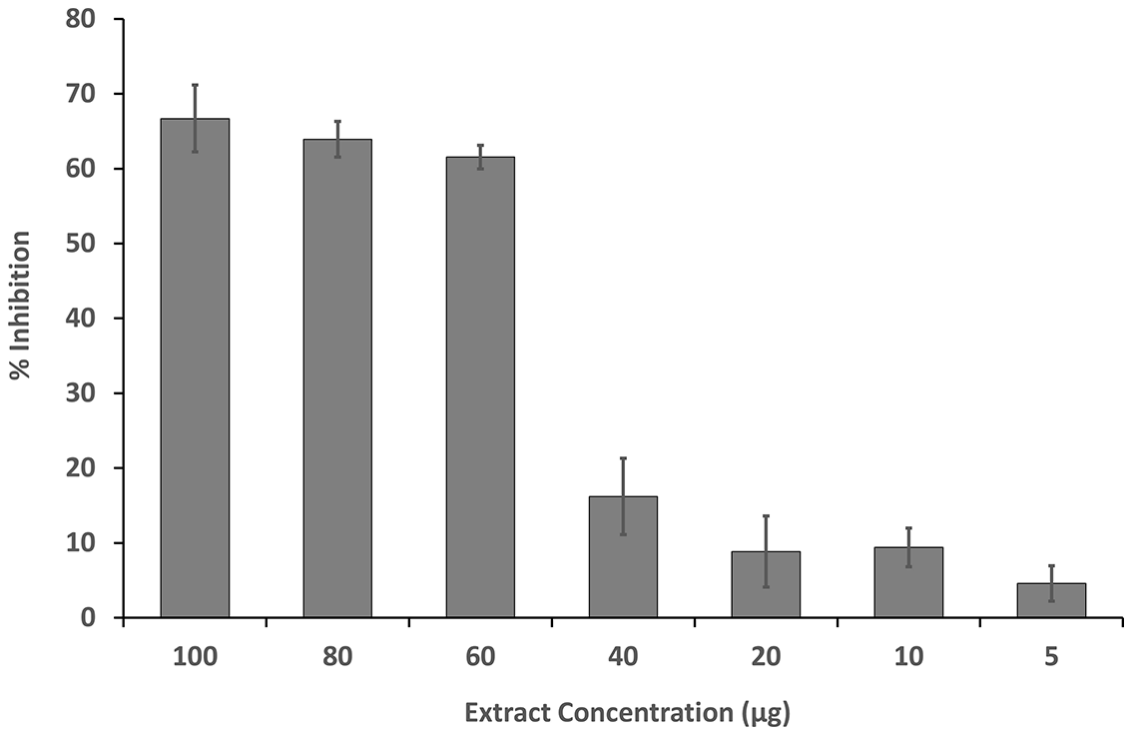
Dose-dependent inhibition of HeLa cells by methanolic fruit extract of *R. semialata* Murray.

The inhibition of HeLa cell growth by the fruit extract was observed in a dose-dependent manner. The IC_50_ of the MExt against HeLa cells was recorded as 61.55 ± 3.37 μg/mL as compared to the standard anticancer compound Doxorubicin (2.37 ± 0.1 μg/mL) (Figure 2).

## Discussion

The edible part of the *Rhus semialata* Murray fruit is its pulp. The pulp contributes 35.39% of the total weight of the fruit. Only the pulp portion was used for analysis in the present study. The proximate analysis of the present study revealed that the fresh fruit contained crude protein (4.25 ± 0.04%), crude fat (19.21 ± 0.76%), total carbohydrate (59.2 ± 1.68%), and ash (4.02 ± 0.13%). Higher amount of protein (8.13 ± 0.38%), lower fat (16.7 ± 0.18%), and carbohydrates (69.2%) content were reported by Loukrakpam et al. (17) in the same fruit, which might be due to the variation in the sample used for analysis (whole fruit including pulp and seed). The mineral composition of the fruit pulp suggested that the most abundant minerals were phosphorous (218 ± 6.8 mg/100 g), iron (19.92 ± 0.2 mg/100 g), and magnesium (14.96 ± 1.2 mg/100 g). These mineral nutrients are required by our bodies to stay healthy. The protein present in this fruit contained both essential and non-essential amino acids. Interestingly, the essential amino acid composition was higher (59.18%) than the nonessential amino acids (40.82%). Essential amino acids are those amino acids that cannot be synthesized by our body and must be present in the human diet. Moreover, the fruit showed significant variation in EAA content ranging from 1.92 to 27.21% of total protein content.

Generally, fruits contain less fat, however *Rhus semialata* Murray fruit was rich in fat (19.21 ± 0.76%). Shi et al. (33) reported a similar amount of fat content (19.68 ± 0.68 to 20.27 ± 1.33%) in *Rhus chinensis* Mill variety. Further analysis of the crude fat revealed that the fruit was rich in polyunsaturated fatty acids. Linoleic acid (C18:2n6c) constituted 92.21% of total PUFA. The essential fatty acids (EFA) such as n6 fatty acids (omega-6 fatty acid) and n-3 fatty acids (omega 3-fatty acid) constituted 49.42 and 1.15% of the total fat of the fruits, respectively. EFA and PUFA derivatives are important structural components of the cellular membrane and play an important role in signal transduction, particularly omega-6 fatty acids. Shi et al. (33) and Loukrakpam et al. (17) also demonstrated high percentage of unsaturated fatty acids in *Rhus chinensis* Mill, and high amount of linoleic acid (C18:2n6c) in *Rhus semialata, respectively*. Hence nutgall fruit is a potential source of unsaturated fatty acids. Unsaturated fatty acids find applications in the food (for formulation of dips and spreads) and pharmaceutical sectors. Most of the phytochemicals (both polar and nonpolar) are dissolved in methanol; hence it represents one of the most used organic solvents for extraction. However, extraction of bioactive metabolites from plants using water as a solvent has been attempted by many researchers. Moreover, the fruit, simply soaked in water, is used as medicine alone or mixed with other ingredients by local traditional healers, especially in Manipur, to treat various ailments. Hence, the fruit pulps were extracted in two solvents, i.e., methanol and water, and screened for biological activities (antioxidant, antihyperglycemic, xanthine oxidase inhibitory, antityrosinase, angiotensin-converting enzyme inhibitory, antibacterial and cytotoxicity of cancer cell lines). Both extracts showed antioxidant activity in all four antioxidant assays. In ABTS and DPPH assay, MExt showed a lower IC_50_ value, which indicates higher antioxidant activity than WExt. Zhang et al. (34) reported that IC_50_ of 80% methanol extract of the *Rhus chinensis* Mill. fruit was 3.72 μg/mL, which is slightly lower than in the studied sample (5.43 ± 0.37 μg/mL). For the FRAP and CUPRAC assay, antioxidant activity was expressed as mM Fe Eq/g Ext and ascorbic acid equivalent (AAE) respectively. The higher the value of mM Fe Eq or AAE, the higher the antioxidant activity. The MExt had higher antioxidant activity than the water extract. Similarly, Sharma et al. (35) reported that the crude methanolic extract of *Rhus semialata* exhibited an IC_50_ value of 5.31 ± 0.07 μg/mL and 5.84 ± 0.17 μg/mL in DPPH and ABTS assays, respectively. The high antioxidant activity of this fruit may be due to the presence of phenolic and flavonoids. These two phytochemicals are known to confer antioxidant activity of many plant extracts. Antioxidant activity is one of the desired properties for the formulation of nutraceuticals or functional foods.

Oxidative stress has been identified as a key factor in the development of diabetic complications (36, 37), and there was a significant association between antioxidant and anti-diabetic activities (38). After observing the potent antioxidant activity of fruit extracts, antihyperglycemic activity was also evaluated. The MExt of fruit showed higher α-amylase inhibitory activity than water extract. We noticed that the MExt and WExt of the fruit were more active against α-glucosidase than α-amylase enzyme. This result demonstrated that the fruit extract has the potential for anti-hyperglycaemic activity in terms of inhibition of α-amylase and α-glucosidase enzymes. Diabetes is closely related to increase production of ROS (reactive oxygen species) and their accumulation via various metabolic pathways (39). In diabetic conditions, as glucose uptake in insulin-dependent tissues (fat and muscle) is minimized, glucose uptake is elevated in insulin-independent tissues (40). This excessive intracellular glucose is converted to polyalcohol sorbitol, resulting in a decrease in the NADPH/NADP+ ratio and glutathione concentration. In addition, hyperglycemia leads to the activation of protein kinase C isoforms, induction of the hexokinase pathway, and overproduction of advanced glycation end products. All these effects of hyperglycemia are responsible for diminishing antioxidant agents and overproducing and accumulating reactive oxygen species, which ultimately leads to oxidative stress (King and Loeken, 2004; Vanessa Fiorentino et al., 2013). Hence, compounds having antihyperglycemic activity as well as antioxidant activity have the potential to be used in controlling diabetes. It has already been reported in the literature that natgall bark extract possesses antihyperglycemic properties. The two compounds responsible of α-glucosidase inhibitory activity have been identified as phloridzin and scopoletin (41). Similarly, Liu et al. (42) studied the inhibitory effect of free phenolic, esterified phenolic and insoluble-bound phenolic from *Rhus chinensis* Mill. on *α*-glucosidase and dipeptidyl peptidase-4 (DPP-IV) and formation of advanced glycation end (AGE) product. They observed that the free phenolic extract had higher *α*-glucosidase and dipeptidyl peptidase-4 inhibitory activity while the insoluble-bound phenolic extract exhibited higher inhibition on AGE product formation. The two phenolic compounds gvajaverin and quercitrin, made the most significant contributions to the inhibitory effects on α-glucosidase and DPP-IV, while trigalloyl glucose and its isomer may be the primary bioactive substances responsible for the suppression of AGE formation (43).

Xanthine oxidase enzyme inhibition is one of the targets for gout and other diseases that involve hyperuricemia (high levels of uric acid in the blood). Hyperuricemia is associated with chemotherapy and calcium oxalate kidney stones in patients. XO inhibitors are a therapeutic option for treating these types of disorders. Current drugs that are available on the market (allopurinol, febuxostat, and uricase, etc.) are associated with adverse side effects (44). Hence, research is ongoing to look for new, effective, and safer XO inhibitors of natural or synthetic origins for the treatment of the disease.

It is a widely known fact that people with diabetes frequently acquire high uric acid levels and vice versa. Desco et al. (45) reported that diabetes causes an increase in xanthine oxidase (XO) activity in the liver. XO catalyzes the oxidation of hypoxanthine to xanthine and xanthine to uric acid (46). Elevated uric acid production can aggravate insulin resistance and lead to diabetes. In addition to uric acid production, XO also plays an important role in generating free radicals, which may influence oxidative damage in diabetes (45, 47). Many antioxidants are also potent xanthine oxidase inhibitors.

In this regard, the *Rhus semialata* Murray fruit extracts were also screened for XO inhibitor activity. The extracts showed inhibitory activity against XO and their IC_50_ (MExt- 93.16 ± 4.65 μg/mL; WExt- 192.2 ± 1.9 μg/mL) was comparable with the standard inhibitor allopurinol (77 ± 0.08 μg/mL). Tsai et al. (48) reported that the n-hexane extract of *Rhus semialata* var. Roxburghiana leaves and stems exhibited xanthine oxidase inhibitory activity. The leaf extract showed higher activity as compared to the stem, as indicated by a lower IC_50_ value. The IC_50_ of leaf extract was recorded as 16.74 ± 0.74 μg/mL, while stem extract was recorded as 26.53 ± 0.54 μg/mL. As far as we know, there are no documentary evidences available on the XO inhibitory activity by fruit extract of *Rhus* *semialata* Murray.

Melanin is produced by the conversion of the amino acid L-tyrosine to 3,4-dihydroxyphenylalanine and its oxidation to dopaquinone (49, 50), the precursor of melanin formation. Tyrosinase catalyzes the conversion of L-tyrosine to melanin. Free radicals also participate in the biosynthesis of melanin and are involved in the catalytic conversion of L-DOPA to dopaquinone by tyrosinase (51). The tyrosinase-inhibiting and free radical scavenging effects of antioxidants inhibit melanin production. Tyrosinase inhibitors are used in the food, pharmaceutical, and cosmeceutical industries. Antioxidants such as vitamin C and E have been shown to have significant inhibitory actions against tyrosinase. Many antioxidant rich fruits have been reported to have antityrosinase activity. Antityrosinase compounds are identified from plant source as well as from microorganisms.

Given this context, the antityrosinase activity of *Rhus semialata* Murray fruit extracts (both MExt and WExt) was screened for the first time in the present study. The crude extracts showed tyrosinase inhibitory activity, though their activity is lesser than the standard kojic acid. It is one of the widely used commercial antityrosinase compounds obtained from different species of fungi and as a byproduct of the fermentation of certain foods (52). Nevertheless, the antityrosinase compound from *Rhus semialata* Murray will also be a potential candidate as a source of antityrosinase compounds.

Angiotensin-converting enzyme I (ACE) inhibitors are a class of drugs used to control high blood pressure. They’re also used to treat other cardiovascular conditions such as heart failure, diabetes-related kidney disease, and more (53, 54). Significant routes leading to hypertension are ROS production in target tissues by the hypertensive agents. These agents stimulate the formation of superoxide through oxidases, uncoupled nitric oxide synthase, xanthine oxidase, and mitochondria (55). ROS can boost the activity of ACE. The oxidation of the sulfhydryl groups on ACE is one possible mechanism. As part of its route, ROS can also activate the renin-angiotensin system (RAS), which includes ACE. ACE is well-known for its twin functions of converting inactive Angiotensin I to active Angiotensin II and degrading active bradykinin, which play a crucial role in blood pressure regulation (56). An increase in angiotensin II production raises blood pressure. Again, several antioxidants possess ACE-inhibiting activities and aid in reducing hypertension.

Though *Rhus semialata* Murray is being used in traditional medicine and shows promising free radical scavenging activity, there is no report on the ACE inhibitory activity of this plant. Hence, the fruit extracts (MExt and WExt) of *Rhus semialata* Murray were assayed for ACE inhibitory activity. The extracts were found to be positive for ACE inhibitory activity, indicating that the fruit exhibits potential antihypertensive activity. The IC_50_ values of MExt and WExt was recorded as 13.35 ± 1.21 μg/mL and 34.57 ± 3.56 μg/mL, respectively. Hence the value-added functional food products prepared using *Rhus semialata* fruit may be used as therapeutic foods in controlling high blood pressure.

Numerous enzymes are responsible for oxidative stress, both directly and indirectly, and the resulting inflammation. For instance, xanthine oxidase increases the generation of superoxide. ROS often stimulates RAS. Several studies have demonstrated an over-activation of the RAS in diabetes problems (57), and the RAS pathway involves ACE. Free radicals are also involved in the tyrosinase-catalyzed conversion of L-DOPA to dopaquinone during melanogenesis. XO, ACE, α-amylase, α-glucosidase, and tyrosinase are therefore related in various ways and contribute to the onset of a number of chronic disorders. *Rhus semialata* Murray, which shows inhibitory capability against these enzymes, might be a significant treatment alternative for degenerative illnesses.

*Rhus chinensis* Mill. was reported to have antibacterial activity against a wide range of gram positive and gram-negative bacteria such as *Bacillus subtilis, Escherichia coli, Klebsiella pneumonia*, *Pseudomonas aeruginosa*, and *Staphylococcus aureus, Streptococcus iniae, Vibrio ichthyoenteri* (58, 59). Similarly, the MExt. of *Rhus semialata* Murray fruit (present study) exhibited antimicrobial activity against *Salmonella typhi* and *Staphylococcus aureus*. However, the extract did not show antibacterial activity against *Bacillus subtilis* or *Escherichia coli*. The variation may be due to the difference in the solvent used for the extraction. Sreedharan et al. (60) also reported that the *Rhus semialata* seed extracted in different organic solvents such as petroleum ether, chloroform, and methanol showed antibacterial activity against at least one of the test organisms (*Klebsiella pneumoniae*, *Staphylococcus aureus*, *Escherichia coli*, *Aspergillus niger* and *Penicillium* sp.). The 80% ethanolic extract of *Rhus javanica* exhibited antibacterial activity against three species of *Shigella, viz. S. sonnei, S. flexneri, S. boydii*, and *S. dysenteriae* (61). These findings indicate that *Rhus semialata* plant extract showed antimicrobial activity against a wide range of gram positive and gram negative pathogenic bacterial genera. However, there is a very limited report on the identification of antimicrobial metabolites from this plant. Hence, further study on the identification of metabolites responsible for antimicrobial activity and their mechanism of action is highly required to discover novel antimicrobial drugs.

Numerous plants have been shown to exhibit high ROS-scavenging activity, which is related with cytotoxicity or antiproliferative activity against cancer cells and might thus be employed as therapeutic and preventative agents (62, 63). The cytotoxicity of *Rhus semialata* against SW620 and HCT116 cell lines has also been reported in the literature. These two cell lines are responsible for colon cancer (64, 65). Taking into account the antiproliferative activity, the MExt of *Rhus semialata* Murray (present study) was evaluated for its *in vitro* cytotoxicity activity against HeLa cell line. It is the oldest and most commonly studied cell lines obtained from cervical cancer specimens. The inhibition of HeLa cell growth by the MExt was observed in a dose-dependent manner until 60 μg/mL concentration corresponding to 60% growth inhibition. Further increases in concentration did not improve the cell growth inhibition significantly. This might be due to an increase in undesired interactions between the compounds present in the extract at higher concentrations, which ultimately retards the activity of the extract. The IC_50_ of the MExt against HeLa cells was recorded as 61.55 ± 3.37 μg/mL. Similarly, Lalawmpuii et al. (66) reported that the methanolic extract of *Rhus javanica* L. fruit exhibited cytotoxicity activity against HeLa cells with an IC_50_ value of 98.28 μg/mL.

## Conclusion

The present study presented thorough scientific evidence for the nutritional and therapeutic potential of *Rhus semialata* Murray grown in North-east Indian Himalaya. Our research showed that fruits are high in minerals and vitamin C. Interestingly, the fruits were also found to be a potential source of essential amino acids, unsaturated fatty acids, omega-6 and omega-3 fatty acids, all of which are vital for maintaining the normal function of the body. The MExt and WExt of *R. semialata* Murray fruits demonstrated multifaceted pharmaceutical properties. Fruit extracts showed antioxidant action equivalent to that of ascorbic acid. Furthermore, the fruit extracts inhibited the α-glucosidase and α-amylase enzyme, indicating antihyperglycemic activity. Besides, the dose-dependent inhibition of HeLa cell growth by the methanolic fruit extract indicated its anticancer potential. To the best of our knowledge, *R. semialta* Murray has been reported for the first time globally for its ACE inhibitory and anti-tyrosinase action. Additionally, the fruits extract exhibited low to moderate pharmacological activity in terms of xanthine oxidase inhibitory and antibacterial activity. The findings of our investigation will provide vital information about the food-value and bioactivity of *R. semialta* Murray fruits. The fruit might be recommended for regular consumption in order to maintain a healthy living. It may also be considered as a component in nutraceutical or functional foods to enhance quality of life or may be researched extensively for innovative value addition. Further investigation into the bioactive compounds responsible for these activities will give deeper insights about this potential fruit.

## Data availability statement

The raw data supporting the conclusions of this article will be made available by the authors, without undue reservation.

## Author contributions

TSS: experimental, data analysis, literature survey, and manuscript drafting. PK: literature survey, experimental, and manuscript drafting. AKD, PL, KT, TC, CD, TBS, SC, and YD: experimental. HS: experimentation and figure and table preparation. RA: literature survey and manuscript drafting. AK: experimental and manuscript drafting. SGS: experimental and language improvement. SKS: data analysis and interpretation. AD: manuscript editing. SR: conceptualization, supervision, interpretation, and final approval of the manuscript. All authors contributed to the article and approved the submitted version.

## Conflict of interest

The authors declare that the research was conducted in the absence of any commercial or financial relationships that could be construed as a potential conflict of interest.

The reviewer HR declared a shared affiliation with the authors SR, TSS, PK, AKD, PL, KT, HS, RA, TC, CD, TBS, SC, YD, SKS, and AD to the handling editor at the time of review.

## Publisher’s note

All claims expressed in this article are solely those of the authors and do not necessarily represent those of their affiliated organizations, or those of the publisher, the editors and the reviewers. Any product that may be evaluated in this article, or claim that may be made by its manufacturer, is not guaranteed or endorsed by the publisher.
